# Deep learning analysis of single‐cell data in empowering clinical implementation

**DOI:** 10.1002/ctm2.950

**Published:** 2022-07-20

**Authors:** Anjun Ma, Juexin Wang, Dong Xu, Qin Ma

**Affiliations:** ^1^ Department of Biomedical Informatics, College of Medicine The Ohio State University Columbus Ohio USA; ^2^ Pelotonia Institute for Immuno‐Oncology, The James Comprehensive Cancer Center The Ohio State University Columbus Ohio USA; ^3^ Department of Electrical Engineering and Computer Science, and Christopher S. Bond Life Sciences Center University of Missouri Columbia Missouri USA

**Keywords:** deep learning, single cell, translational study

Recent advances in single‐cell sequencing technologies enable the characterization of cellular heterogeneity and biological processes in complex diseases. This provides unprecedented opportunities to understand disease pathology at a level that allows mechanistic classification and development of precision therapeutic strategies. Extensive research has been performed in clinical studies at the single‐cell level.^1^ In addition, emerging deep learning (DL) technologies hold great potential in modeling large‐volume and highly heterogeneous single‐cell data by using sophisticated architectures, such as artificial neural networks,^2^ for translational and clinical purpose.^3^ In this commentary, we focus on the DL analysis of single‐cell data in empowering the clinical implementation of personalized medicine.

## 1 DEVELOPING DIAGNOSIS METHODS

Single‐cell technology (e.g., single‐cell RNA sequencing [scRNA‐seq]) for characterizing diseased cell populations was first applied to cancers and then to Alzheimer disease and chronic bowel disease.^4^, ^5^ DL technologies can extract and recognize features from single‐cell data in a hypothesis‐free manner, especially neglected and inconspicuous features in cell subpopulations, such as clonal tumor subtypes, minimal residual disease (MRD), and cancer stem cells (CSCs). These cells are critical in disease treatment and vulnerable to evolution, but they represent only a tiny proportion in samples, while maintaining high heterogeneity among patients. Identifying clonal tumor subtypes characterizes tumor heterogeneity and significantly improves disease prognosis. The DL framework RDAClone was used with an extended robust deep autoencoder to embed noisy single‐cell genomics sequencing data in order to cluster cells into subclones and infer subclone evolutionary relationships.^6^ Another hybrid deep clustering approach was used to identify potential tumor subclones in triple‐negative breast cancer samples and investigate the role of clonal heterogeneity.^7^

MRD plays a pivotal role in the initiation and progression of diseases, such as cancer. However, studying small tissue samples and rare cell populations is a major challenge in efficient translational studies of MRD. To successfully detect the presence of certain rare cell populations, researchers used a DL model trained from known cell populations in large‐scale cell atlas studies, combined with single‐cell sequencing, to adopt deep transfer learning and transfer the knowledge to unseen MRD data.^8^

CSCs, a subpopulation of tumor cells, drive tumor growth and give rise to differentiated progeny. Targeting genes specific to CSCs may have therapeutic potential. For example, DeepCpG, a deep neural network (DNN)‐based computational approach, applies modular DL architecture to learn features from single‐cell bisulfite sequencing data.^9^ In DeepCpG, the DNA module consists of two convolutional and pooling layers to identify predictive motifs from the local sequence context and one fully connected layer to model motif interactions; the CpG module scans the CpG status in multiple cells using a bidirectional Gated Recurrent Unit (GRU) neural network; and the joint module learns interactions between higher‐level features derived from the DNA and CpG modules to predict methylation states in all cells. DeepCpG can be used to differentiate human induced pluripotent stem cells (iPSCs) in parallel with transcriptome sequencing in order to specify splicing variation (exon skipping) and its determinants. The scVI tool uses stochastic optimization, variational autoencoders, and generative modeling to compute cell embeddings and gene expression distribution. It enables multiple analysis, including batch effect removal, cell cluster prediction, gene imputation, and differentially expressed gene identification.^10^ scVI can identify CSC populations and determine what types of cells CSCs can differentiate into. This way, stem cell subsets with the required differentiation direction can be directly used for treatment. Similar DL technologies may be used to detect circulating tumor cells (CTCs, isolated tumor cells entering the circulatory system of a patient with cancer), which are considered an effective tool for diagnosing malignancy.

## 2 ASSISTING DISEASE MECHANISM STUDIES

DL technologies can be especially used to model single‐cell sequencing data in order to determine the underlying molecular mechanisms in immuno‐oncological microenvironment. We applied a heterogeneous graph transformer model to specific gene regulatory networks in two abnormal B‐cell stages from diffusing small lymphocytic lymphoma samples by integrating scRNA‐seq and single‐cell assay for transposase‐accessible chromatin with sequencing (scATAC‐seq) data.^11^ Analysis of scRNA‐seq samples before and after treatment may reveal subsets refractory to a given therapy and their biomarkers and mechanism response to immune‐checkpoint therapy (ICT).^1^ scRNA‐seq shows that the effects of different ICTs on monocytes/macrophages in tumors are especially significant, leading to a high degree of plasticity and complexity in the cell population.^12^ DeepGeneX uses a two‐phase DNN to predict a patient's response to immunotherapy. First, it removes genes that are less important to response prediction according to gene permutation, and then, it predicts the responsiveness of the patient using a fully connected layer based on the remaining highly important genes. Studies have used DeepGeneX to identify high *LGALS1* and *WARS* expression in macrophage populations as a biomarker for ICT nonresponders, indicating that these macrophages may be a target for improving ICT response.^13^

## 3 SUPPORTING DRUG DESIGN

Another emerging clinical application of DL technologies at the single‐cell level is drug‐related predictions, such as drug response, drug repurposing, and drug combination.^14^ DL models have been used for drug‐related predictions at the bulk level for years,^14^ yet research at the single‐cell level is still in its infancy due to insufficient training data in the public domain. Massive bulk gene expression databases incorporating drug‐screening data can be used to determine the optimal clinical application of cancer drugs. Intuitively, drug‐related bulk RNA‐seq data may help infer gene expression–drug response relationships and predict drug responses at the single‐cell level. Deep transfer learning can transfer knowledge and relationship patterns from bulk data to single‐cell data to overcome the issue of limited training data.^15^ scDEAL, a deep transfer learning framework integrating large‐scale bulk and scRNA‐seq data, adapts a domain‐adaptive neural network to predict single‐cell drug responses from scRNA‐seq data by integrating and harmonizing large‐scale drug response data of bulk cancer cell lines; it does not depend on predefined single‐cell labels.^16^ It can further predict critical genes that significantly contribute to drug sensitivity and resistance prediction. In another study, a convolutional neural network (CNN)‐based model was designed to predict antitumor drugs for CTCs at the single‐cell level.^17^ Analysis of single‐cell subsets identified a combination therapy that targeted two mutually exclusive pathways, more effective than monotherapy, in a patient‐derived xenograft model. Single‐cell DL analysis may also be used for drug repurposing^18^ and drug design^19^ for patients with infections during the coronavirus disease 2019 (COVID‐19) pandemic.

## 4 CHALLENGES AND PERSPECTIVES

With the development of single‐cell and DL technologies, we can foresee broad DL applications in clinical studies at the single‐cell level. A pioneer practice led by the LifeTime Initiative aims to track, understand, and target human cells during the onset and progression of complex diseases and to analyze their response to therapy using DL at the single‐cell level.^20^ In addition to existing DL methods, such as CNNs, deep transfer learning, and graph neural networks, many advanced DL frameworks hold great potential. For example, meta‐learning^21^ and few‐shot learning^22^ strategies can help improve model generality by combining abundant public cell atlas and rich clinical‐specific data from patients’ electronic health records. Knowledge‐based neural networks,^23^ which construct DL architectures using known biological data, can help make single‐cell DL analysis more biologically relevant and explainable. Emerging federated learning strategies may support DL models across multiple decentralized servers holding local data.^24^

The challenges limiting DL's clinical applications at the single‐cell level are as follows: Clinical and Translational Medicine

1. Limited availability of single‐cell sequencing data in clinical studies. The isolated, private, and sparse patient data collected in diverse quality and formats from different institutions are usually difficult to access and are handled by classical DL methods designed for basic research. Clinical practitioners need to be more proactive in collecting patient data and provide them for research.
2. Limitations of current DL models’ capacities in transferring knowledge from basic research to clinical research. DL models, which are designed and trained on public atlas single‐cell sequencing data, often do not work well in individual, patient‐specific studies in clinical practice. Extensive method development is required to make DL models generalizable, robust, and explainable.
3. Limited availability of benchmarks for DL models developed in clinical research. Unlike basic benchmark studies that can use a large amount of public data, few golden‐standard data exist for clinical studies.^25^ The research community needs to develop data standards and make data DL‐ready.

In summary, accumulation of high‐quality single‐cell sequencing data in both basic and clinical research fosters the development of DL algorithms and their applications in new areas. The growth of DL modeling with the availability of fine‐grained, cell‐based clinical sequencing data pushes the understanding, diagnosis, and treatment of diseases in clinical practice. With the maturation of single‐cell technologies in clinical research and the continuous advancements in DL, more translational clinical applications can be developed.

## CONFLICT OF INTEREST

The authors declare that there is no conflict of interest that could be perceived as prejudicing the impartiality of the research reported.
